# Supplementary material for: Effect of Adhesion and Substrate Elasticity on Neutrophil Extracellular Trap Formation
Source: Front Immunol. 2019 Oct 1;10:2320. doi: 10.3389/fimmu.2019.02320 (PMC6781793; doi:10.3389/fimmu.2019.02320)
Supplement: Supplementary file 1 [file Data_Sheet_1.pdf]

Supplementary information

Effect of Adhesion and Substrate Elasticity on Neutrophil Extracellular Trap Formation

Luise Erpenbeck[1#*], Antonia Luise Gruhn[1#], Galina Kudryasheva[2], Gökhan Günay[1,4], Daniel Meyer[4], Julia Busse[1], Elsa Neubert[1, 4], Michael P. Schön[1,3], Florian Rehfeldt[2], Sebastian Kruss[4*]

[1]*Department of Dermatology, Venereology and Allergology, University Medical Center, Göttingen University, Germany*

[2]*Third Institute of Physics - Biophysics, Göttingen University, Germany*

[3]*Lower Saxony Institute of Occupational Dermatology, University Medical Center Göttingen, Germany*

[4]*Institute of Physical Chemistry, Göttingen University, Germany*

[#]*These authors contributed equally*

*Corresponding authors: skruss@gwdg.de (SK), luise.erpenbeck@med.uni-goettingen.de (LE)

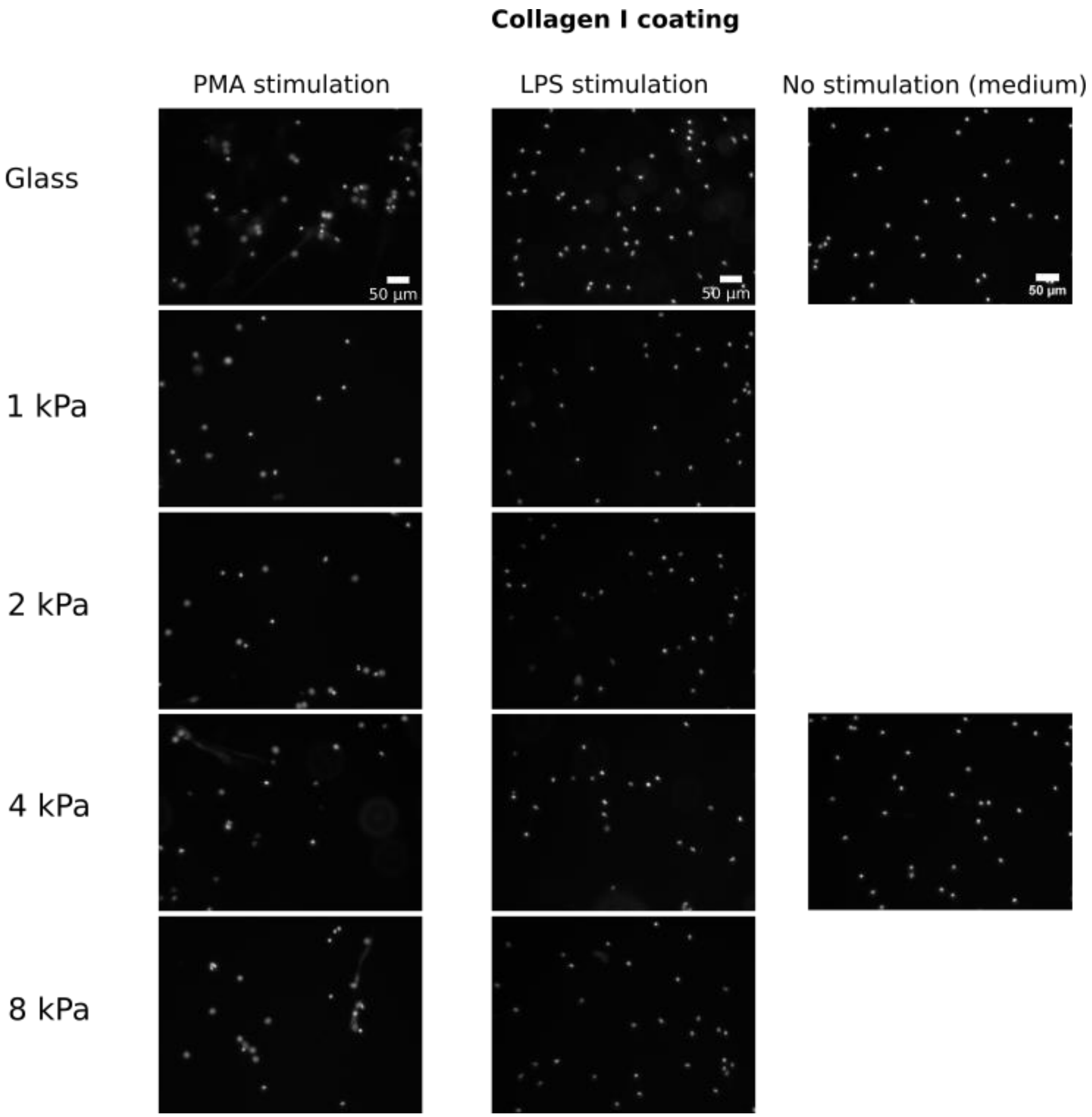
Collagen I coating
PMA stimulation
LPS stimulation
No stimulation (medium)
Glass
1 kPa
2 kPa
4 kPa
8 kPa
50 µm
50 µm
50 µm

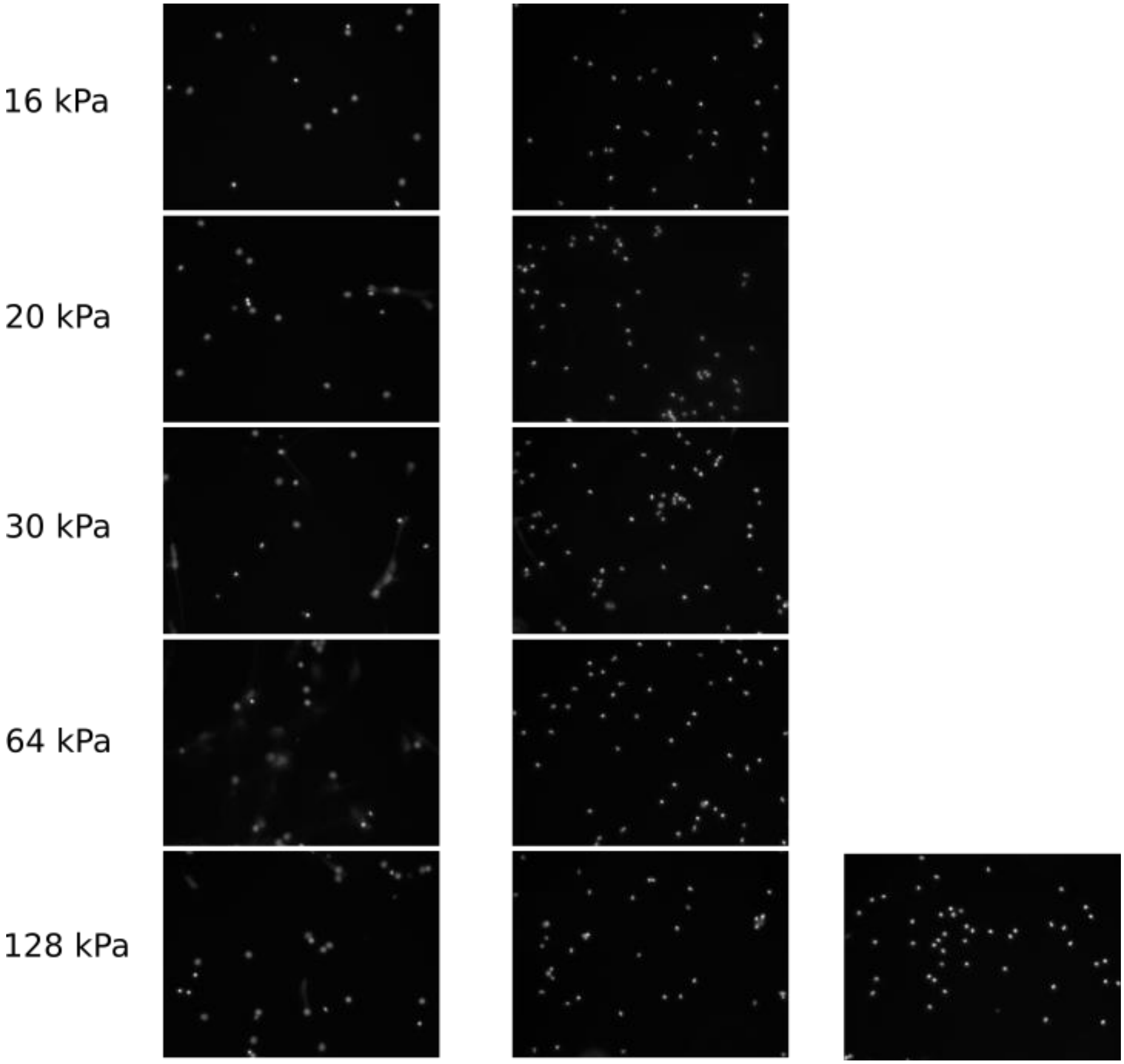

Supplementary figure S1. ***Fluorescence images of stimulated neutrophils on PAA gels coated with collagen I.*** *Freshly isolated neutrophils were seeded on polyacrylamide substrates and glass surfaces coated with collagen I and stimulated either with PMA or LPS for 3h. As vehicle control, neutrophils on 4 kPa and 128 kPa gels were incubated only with RPMI media. After fixation with 2% PFA, cells were stained with Hoechst 33342/ DNA and representative images were taken from each well to quantify NETosis rates.*

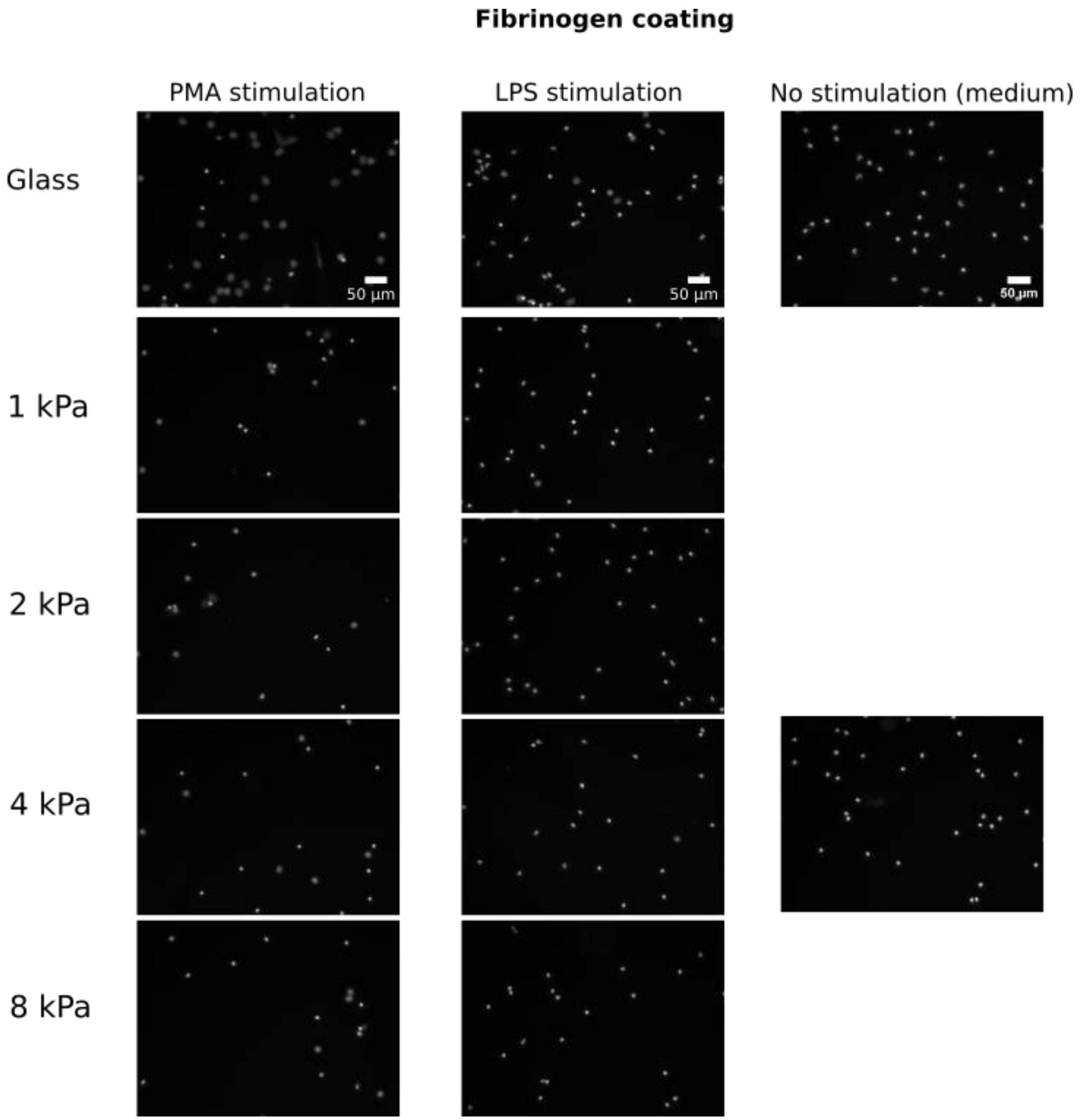
Fibrinogen coating
PMA stimulation
LPS stimulation
No stimulation (medium)
Glass
1 kPa
2 kPa
4 kPa
8 kPa
50 µm
50 µm
50 µm

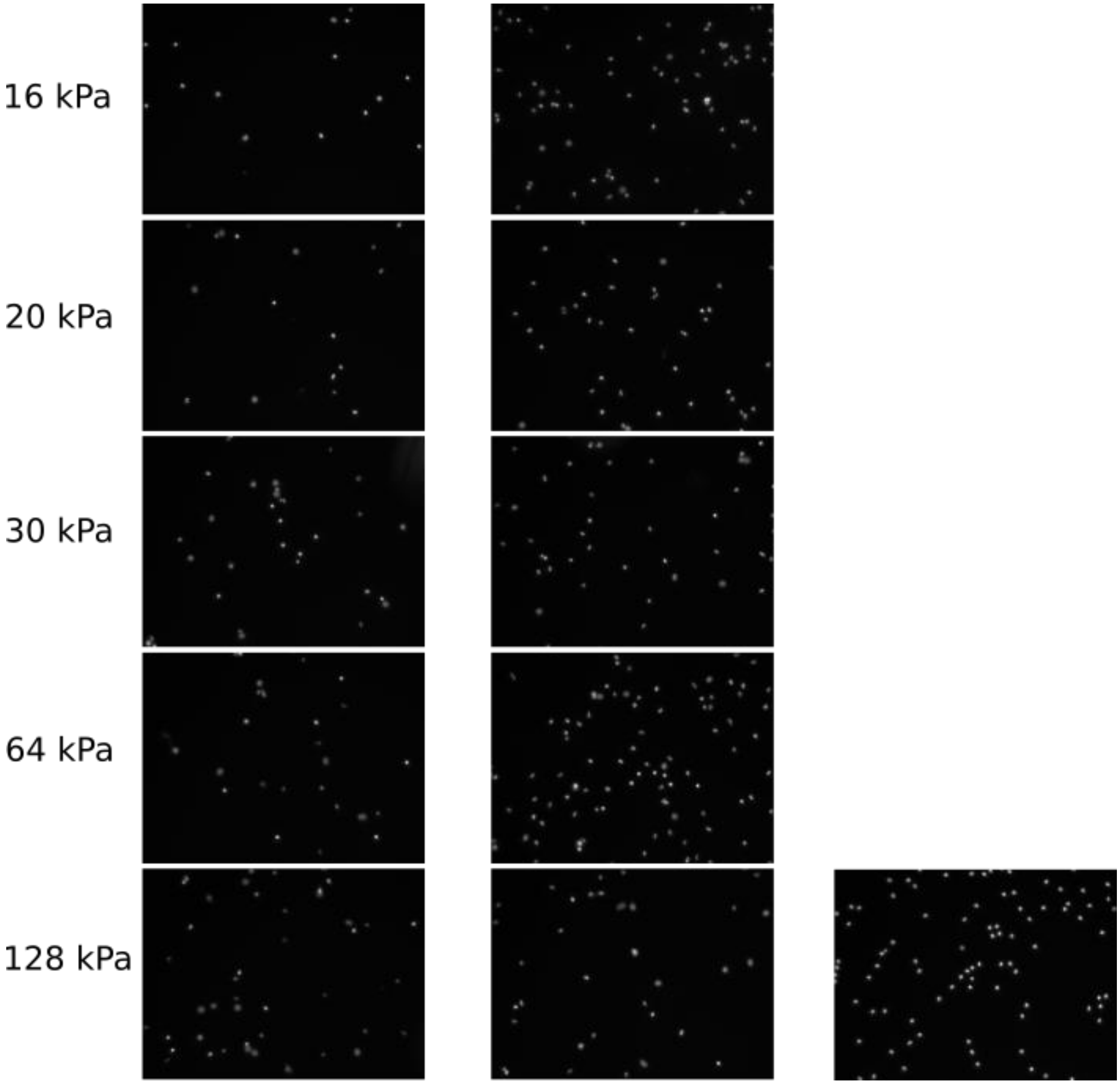

Supplementary Figure S2 ***Fluorescence images of stimulated neutrophils on PAA gels coated with fibrinogen*.** *Freshly isolated neutrophils were seeded on polyacrylamide substrates and glass surfaces coated with fibrinogen and stimulated either with PMA or LPS for 3h. As vehicle control, neutrophils on 4 kPa and 128 kPa gels were incubated only with RPMI media. After fixation with 2% PFA, cells were stained with Hoechst 33342/ DNA and representative images were taken from each well to quantify NETosis rates.*

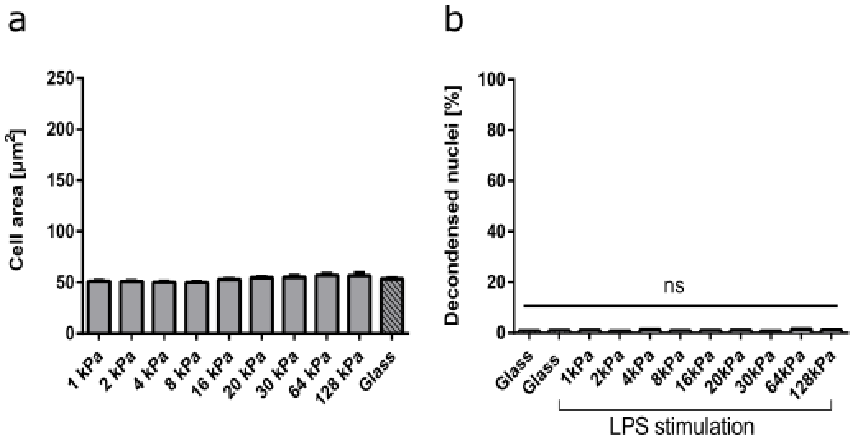

Supplementary figure S3: NETosis on PAA gels functionalized with higher collagen concentrations. PAA gels of different elasticity were functionalized with a tenfold higher collagen I concentration (0.2 mg/ml). On those gels neutrophils did not properly adhere and spread (a). Consequently, LPS-induced NETosis did not occur (b).

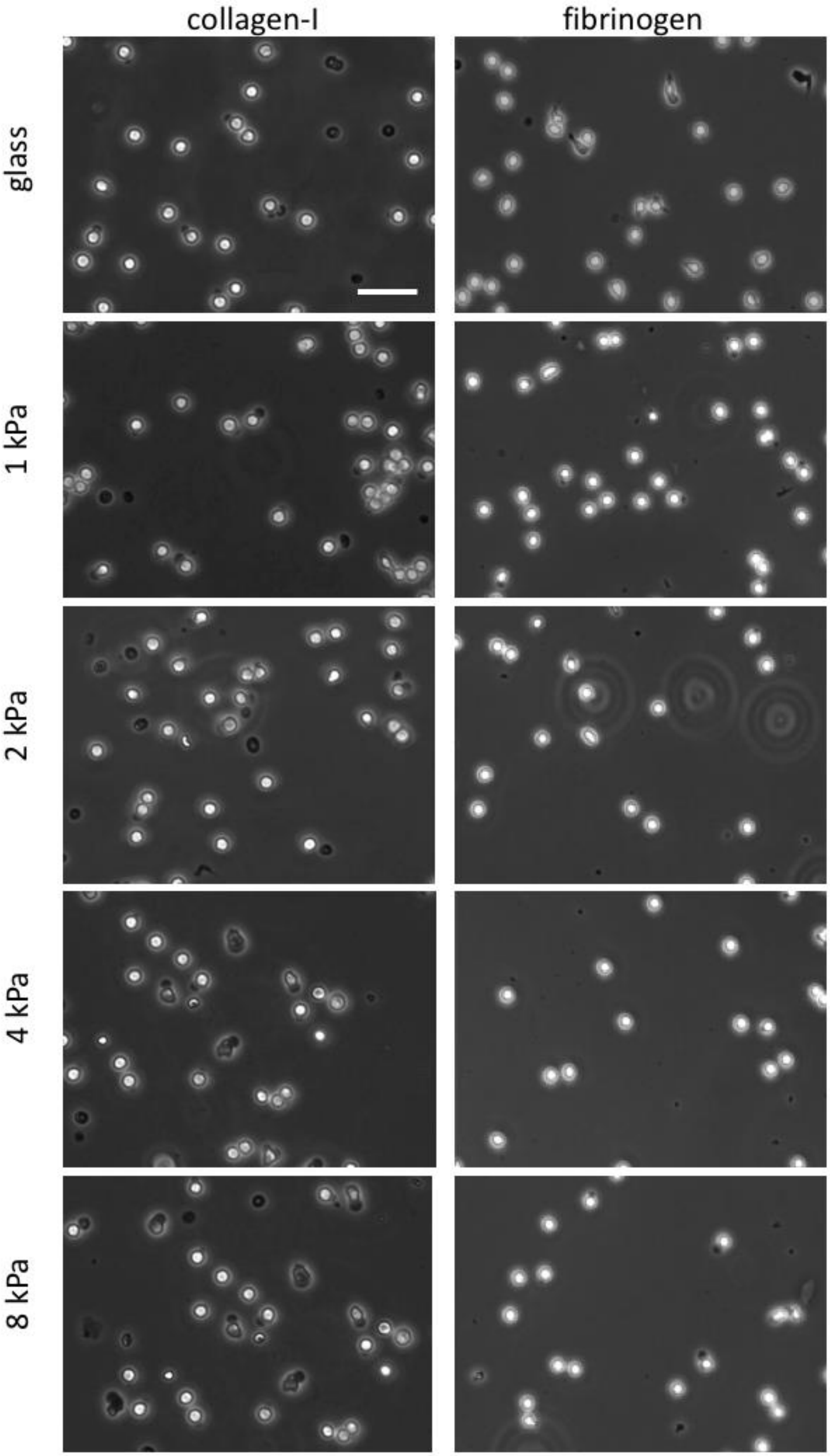
collagen-I
fibrinogen
glass
1 kPa
2 kPa
4 kPa
8 kPa

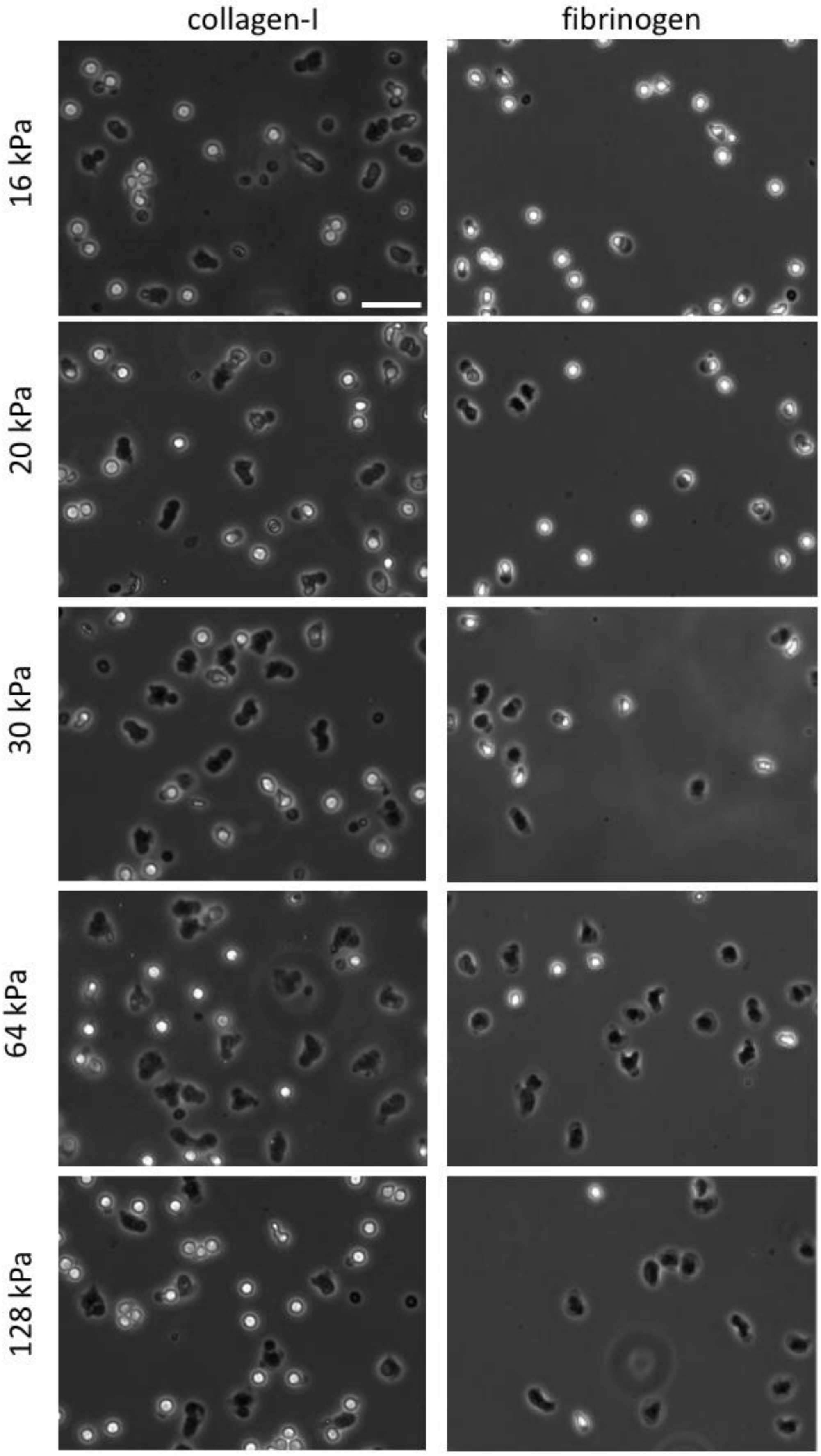
collagen-I
fibrinogen
16 kPa
20 kPa
30 kPa
64 kPa
128 kPa

Supplementary Figure S4 ***Phase contrast images of unstimulated neutrophils on PAA gels coated with either collagen-I or fibrinogen.*** *Freshly isolated neutrophils were seeded on polyacrylamide substrates and glass surfaces coated with collagen-I or fibrinogen. After fixation with 2% PFA representative images were taken from each well to quantify changes in cell area.*

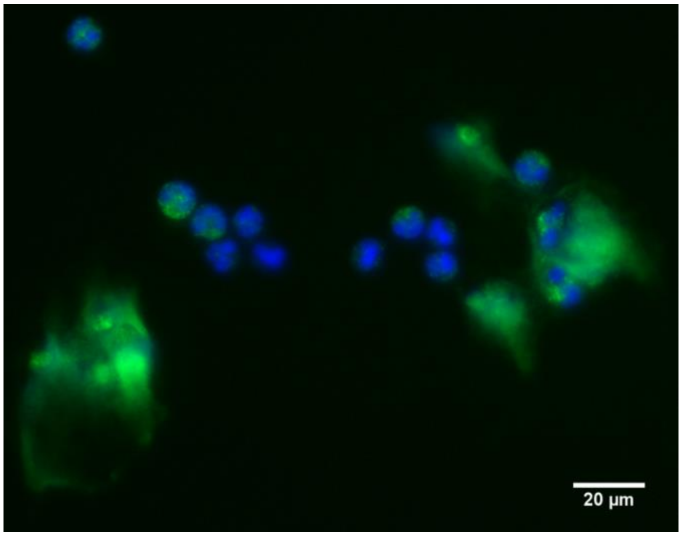

Supplementary Figure S5 *Neutrophils were seeded on a collagen I-coated 128 kPa PAA gel and stimulated by LPS as described above. After fixation with PFA, cells were stained with anti-MPO antibody (green) and Hoechst against chromatin (blue) and imaged by conventional immunofluorescence microscopy to verify NET production.*

Table T1: Composition of the PAA gels and Young's modulus

Young's modulus *E* [kPa]	%(v/v) acrylamide in PBS	% (v/v) bis-acrylamide in PBS
1	3	0.20
2	3.5	0.20
4	3.8	0.20
8	6.8	0.10
16	6.8	0.20
20	8	0.14
32	8.6	0.30
64	13.2	0.30
128	23.6	0.30
